# Neurological Sequelae Resulting from Encephalitic Alphavirus Infection

**DOI:** 10.3389/fmicb.2016.00959

**Published:** 2016-06-20

**Authors:** Shannon E. Ronca, Kelly T. Dineley, Slobodan Paessler

**Affiliations:** ^1^Department of Pathology, University of Texas Medical Branch, Galveston, TXUSA; ^2^Department of Preventive Medicine and Community Health, University of Texas Medical Branch, Galveston, TXUSA; ^3^Department of Neurology, Center for Addiction Research, Rodent In Vivo Assessment Core, Mitchell Center for Neurodegenerative Disorders, University of Texas Medical Branch, Galveston, TXUSA; ^4^Institute for Human Infections and Immunity, Galveston National Laboratory, University of Texas Medical Branch, Galveston, TXUSA

**Keywords:** alphavirus, sequelae, behavior, VEEV, WEEV, VEEV

## Abstract

The recent surge in viral clinical cases and associated neurological deficits have reminded us that viral infections can lead to detrimental, long-term effects, termed sequelae, in survivors. Alphaviruses are enveloped, single-stranded positive-sense RNA viruses in the Togaviridae family. Transmission of alphaviruses between and within species occurs mainly via the bite of an infected mosquito bite, giving alphaviruses a place among arboviruses, or arthropod-borne viruses. Alphaviruses are found throughout the world and typically cause arthralgic or encephalitic disease in infected humans. Originally detected in the 1930s, today the major encephalitic viruses include Venezuelan, Western, and Eastern equine encephalitis viruses (VEEV, WEEV, and EEEV, respectively). VEEV, WEEV, and EEEV are endemic to the Americas and are important human pathogens, leading to thousands of human infections each year. Despite awareness of these viruses for nearly 100 years, we possess little mechanistic understanding regarding the complications (sequelae) that emerge after resolution of acute infection. Neurological sequelae are those complications involving damage to the central nervous system that results in cognitive, sensory, or motor deficits that may also manifest as emotional instability and seizures in the most severe cases. This article serves to provide an overview of clinical cases documented in the past century as well as a summary of the reported neurological sequelae due to VEEV, WEEV, and EEEV infection. We conclude with a treatise on the utility of, and practical considerations for animal models applied to the problem of neurological sequelae of viral encephalopathies in order to decipher mechanisms and interventional strategies.

## Introduction

Alphaviruses are a genus of family *Togaviridae*. There are 29 recognized species of alphaviruses. Alphaviruses are positive sense single-stranded RNA viruses that commonly cause febrile illness followed by either encephalitic or arthralgic disease. Alphaviruses are grouped by the composition of their antigenic complexes. At least eight antigenic complexes have been described (Calisher et al., 1973, 1980; Boere et al., 1984; Greiser-Wilke et al., 1989; reviewed in Griffin, 2007). The arthralgic alphaviruses are designated Old World alphaviruses and include Sindbis virus (SINV), Chikungunya virus (CHIKV), and many other members of the Semliki forest (SF) antigen complex. The encephalitic viruses are characterized as New World alphaviruses that have evolved separately from Old World alphaviruses and include Venezuelan equine encephalitis virus (VEEV), Eastern equine encephalitis virus (EEEV), and Western equine encephalitis virus (WEEV) (Calisher et al., 1980; reviewed in Griffin, 2007; Forrester et al., 2012; Voss et al., 2014).

**FIGURE 1:**
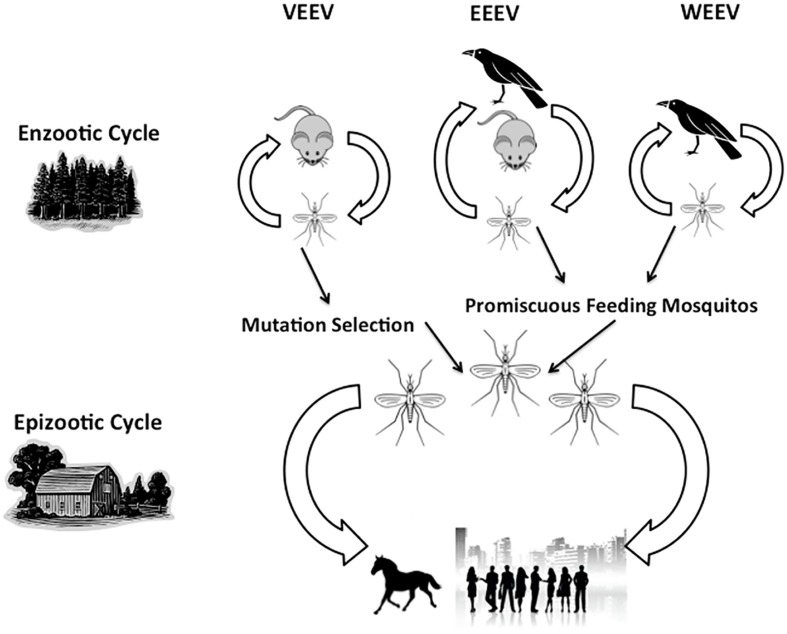
**Transmission cycles of Venezuelan equine encephalitis viruses (VEEV), Western equine encephalitis viruses (EEEV), and Eastern equine encephalitis viruses (WEEV).** The virus naturally occurs in the enzootic cycle as it shifts between the animal host (rodent or bird) and the mosquito vector. Other mosquitos can feed on infected animal hosts, thus transmitting the virus to humans, horses, and other large mammalian hosts, leading to an epizootic cycle.

The bite from an infected mosquito transmits alphaviruses in the enzootic and epizootic cycles (**Figure 1**). Thus, the species of mosquito varies for each alphavirus and determines several aspects of the biology of transmission and etiology of disease including the mechanism of viral maintenance in natural reservoirs to the type of infection caused in humans and other hosts (Kesler, 1933; Kubes and Rios, 1939; Suarez and Bergold, 1968). Typically, infection with VEEV, WEEV, and EEEV leads to a febrile illness following a 1–14 day incubation period (CDC, 2012, 2013). **Table 1** summarizes these virus-specific epidemiological and clinical details. Currently, there are no licensed vaccines in the United States, but live-attenuated and formalin-inactivated version of VEEV, EEEV, and WEEV have previously been used in the US military and laboratory workers. However, 15–30% of vaccine recipients develop febrile symptoms (Tigertt et al., 1962; McKinney et al., 1963; Alevizatos et al., 1967; reviewed in Griffin, 2007). Unfortunately, there are no specific therapeutic interventions; as such, clinicians are limited to palliative care and supportive treatment for alphavirus-infected patients.

**Table 1:** Encephalitic alphaviruses.

	Venezuelan encephalitis viruses (VEEV)	Western encephalitis viruses (WEEV)	Eastern equine encephalitis viruses (EEEV)
Transmission	Rodents, mosquitos (*Aedes, Culex*, *Psorophora* etc.)	Birds, mosquitos (*Aedes, Culex*, etc.)	Rodents/birds, mosquito (*Aedes, Culiesta, etc.)*
Average incubation time	1–5 days	2–7 days	3–10 days
Initial symptoms	Asymptomatic, febrile illness	Asymptomatic, febrile illness	Asymptomatic, febrile illness
Clinical syndrome	Encephalitis	Encephalitis	Encephalitis
Case-fatality (%)	1	3–7	50–75
Associated neurological sequelae	Convulsions, somnolence, confusion, photophobia, coma, intellectual disability, and emotional instability/behavioral changes	Confusion, visual disturbances, photophobia, seizures, somnolence, coma, intellectual disability, and emotional instability/behavioral changes, and spastic paresis	Convulsions, seizures, paralysis, intellectual disability, and behavioral changes
% Survivors with neurological sequelae	4–14	15–30	50–90

Outbreak records and case reports from the past century indicate that neurological sequelae are primarily associated with VEEV, WEEV, and EEEV infection; although recent studies report sensorineural hearing loss associated with CHIKV infection (Rampal et al., 2007; Bhavana et al., 2008; Chusri et al., 2011). Thanks to improved supportive therapy with modern medical technology, many patients now survive the acute phase of brain infection, yet go on to develop permanent neurological sequelae that greatly reduce the quality of life of survivors and their caregivers in addition to placing a high financial burden on them and our healthcare system. For example, treatment of one person suffering residual sequelae from EEEV costs approximately $4.6M in lifetime care (Villari et al., 1995). Even patients whose acute illness is relatively mild, frequently have persistent movement disorders, cognitive complaints, and functional disability (Mulder et al., 1951; Finley and Chapman, 1953; Fulton and Burton, 1953; Palmer and Finley, 1956; Hanson, 1957; Herzon et al., 1957; Bowen et al., 1976; Deaton et al., 1986). Therefore, vaccines for prevention or interventions during or post-acute illness could have significant impact on the quality of life as well as on the healthcare cost.

## Clinical Cases

The term ‘sequelae’ is broadly defined as a condition resultant from previous disease. Neurological sequelae are those complications involving the central nervous system (CNS) that include cognitive, sensory, and motor deficits that may encompass emotional instability and seizure activity in the most severe cases. Clinical case studies have documented that infection with either VEEV, WEEV, or EEEV can cause severe neurological sequelae, such as neuropsychological changes and intellectual disabilities (**Table 1**). A variety of animal models exist to study alphavirus encephalitis including mice, hamsters, guinea pigs, rabbits, equines, and nonhuman primates (reviewed in Nalca et al., 2003; Steele and Twenhafel, 2010; Zacks and Paessler, 2010). For each of these models, there are studies to describe these animal models and the potentially associated viral and immunological factors that could contribute to lethality and neuroinvasion, but it is difficult to extrapolate the information from these studies without knowing how these models would present neurological sequelae. Human clinical cases are our best documentation of neurological sequelae and the best glimpse into potential mechanisms. Moreover, records for these diseases in some areas of the world are incomplete and often categorized with other similar diseases, so the human data is not a true reflection of the incidence, severity, or characteristics of alphavirus encephalitis. In fact, the World Health Organization (WHO) does not track these diseases independently, but rather refers to them as viral encephalitis, with a focus on West Nile Virus and Japanese Encephalitis, making it difficult to establish the full realm of cases with sequelae. Thus, limitations of the clinical data further force us to depend on the large outbreak cases described in the early 1950s, 60s, 70s, and 80s.

### Venezuelan Equine Encephalitis

The number of cases of VEE reported each year is relatively low compared to the other alphaviruses, although we would argue this is a result of misdiagnosis of other arboviral encephalitis agents. VEEV was first isolated in 1936 in equines (Kubes and Rios, 1939) but it was not until the 1960s when human cases occurred in various regions of Venezuela that the virus was identified as the cause of human disease. Little data is available regarding the neurological complications of these original cases, but studies at the time were able to confirm the role of *Aedes* mosquitos in transmitting the virus in Venezuelan urban areas (Suarez and Bergold, 1968). Although the Centers for Disease Control and Prevention (CDC) have documented cases of VEEV infection in the US, little information is available beyond case numbers and deaths. For example, in 1971, a VEEV outbreak occurred in Texas that reported 86 hospital-based surveillance cases. Forty-eight of the 86 cases reported follow up nine months to one year after recovery. Of these, 12 cases were confirmed to present with long-lasting neurological sequelae occurring months after the clearance of the acute infection. In these cases, sequelae included paralysis, reductions in hearing, taste, and smell, as well as recurrent headaches, severe fatigue, and depression (Bowen et al., 1976). In 1995, a large outbreak occurred in Columbia, leaving 75,000 infected, 300 dead, and 3,000 with neurological complications. Seizures and neuropsychological changes were common among those with neurological complications (Rivas et al., 1997). More recently, in 2010, a Panamanian VEEV outbreak occurred alongside EEEV, during which 11 confirmed cases of VEE were reported, four of which experienced long-term sequelae. Although these patients were young males (between the ages of 3–18) and spent less than one week in the hospital, three patients suffered altered mental status and one experienced seizures (Carrera et al., 2013). In spite of VEE being historically underreported, we cannot rule it out as a large contributor to human encephalitic disease with resultant sequelae.

### Western Equine Encephalitis

Similar to VEEV, WEEV was first isolated in 1930 (Meyer et al., 1931) and *Culex* mosquito transmission was confirmed in 1933 (Kesler, 1933). Between 1964 and 2010, 640 cases of WEE were reported in the United States by the CDC (2010). Cases have dwindled over the past few years for as yet to be determined reasons, but a drop in viral virulence is not considered to underlie this trend (Forrester et al., 2008). Although the mortality rate for WEE is low, upwards of 30% of infections develop neurological sequelae. As such, WEE cases provide us with the majority of clinical data regarding sequelae. This can provide insight into common mechanisms between the three closely related encephalitic alphaviruses.

Between 1939 and 1956, there were 636 recorded cases of WEE in the United States and Canada, of which 86 cases had sequelae. The sequelae included brain damage leading to quadriplegia, hemiplegia, and intellectual disability. The frequency of these sequelae in children under one year of age was greater than 50%. In adults, a Parkinson-like disease was reported, as well as schizophrenia-like personality changes (Herzon et al., 1957). Fifteen of these cases from the 1949 outbreak were followed for 6–8 months after acute illness recovery (Mulder et al., 1951) and the 1952 epidemic of viral encephalitis including both WEE and St Louis Encephalitis in California (Palmer and Finley, 1956). In both outbreaks, patients were evaluated by a psychiatrist and subjected to age-specific psychological and cognitive tests. Seizures, decreased motor skills, intellectual disability, learning disabilities, speech difficulty, psychological impairment, altered gait, taste distortion, and loss of facial movements were reported in children and adults although both studies concluded higher incidence of sequelae in children (Mulder et al., 1951; Palmer and Finley, 1956). Interestingly, Palmer et al. noticed that children under 3 months of age seemingly recovered from the sequelae over time, but these children then experienced similar sequelae months or years following the described recovery (Palmer and Finley, 1956).

Similar to the findings of Palmer, Mulder, and Herzon, another research group performed a follow up study for a Texas outbreak that included 35 WEEV cases between 1963 and 1966 (Earnest et al., 1971). Follow up occurred 2–7 years after acute infection. Of the 22 cases available for follow up, 12 children and 3 adults had measurable sequelae. The researchers divided the sequelae into three categories based on psychological and cognitive sequelae. The majority of patients (seven) experienced intellectual disability, seizures, and spastic weakness, one had hearing and speech deficit, and four had minimal brain dysfunction, such as attention deficit disorder (Earnest et al., 1971). This distribution of sequelae is typical for these alphaviruses.

Interestingly, two separate case studies twenty years apart describe overlapping sequelae. In 1962, Cohn and Kuida described the case of a 43-year old male infected with WEEV who previously had no respiratory symptoms. After infection, he developed primary alveolar hypoventilation, a very rare condition that can be due to damaged motor neurons (Cohn and Kuida, 1962). In 1981, a 17 year-old male recently infected with WEEV was found to have the same symptoms and diagnosis. In this case, the young male also developed sleep apnea. His condition was treatable with chemical stimuli and over a period of 3 to 4 months his condition improved (White et al., 1983).

Arguably, the most devastating of the sequelae are those related to severe neuropsychological changes. Depression, anxiety, paranoia, and decreased intellectual abilities were present in many of the cases discussed above (Mulder et al., 1951; Palmer and Finley, 1956; Herzon et al., 1957). A psychological study of alphavirus patients in Canada (Fulton and Burton, 1953) led researchers to conclude that psychological patients in hospital facilities may be suffering the sequelae of alphavirus encephalitis, yet were wrongly diagnosed with psychiatric disorders since these patients were unresponsive to conventional treatments for their diagnoses. Tests in two institutional hospitals supported this concept, as 36 of the patients tested were confirmed to have WEEV antibodies, while seven additional patients were suspected to have had viral encephalitis, although antibody tests were inconclusive (Fulton and Burton, 1953).

A particularly devastating example of misdiagnosis emanates from a 1986 case report from Deaton and colleagues followed the case of a 22-year-old female psychiatric patient, later determined to be the sequelae of an encephalitic infection. The patient presented with psychotic and neuropsychological symptoms, such as severe mood swings, paranoia, generalized confusion, and decreased ability to perform daily tasks, including her job as a cashier. Her family had her evaluated by a psychiatrist under the belief that her symptoms were related to emotional stress. The psychiatrist diagnosed her as “acutely psychotic” and had her institutionalized. Initially, she was treated with phenothiazines, which target G-protein coupled receptors such as dopamine and andregenic receptors, and electroconvulsive shock therapy, which alters the brain chemistry. Rapidly, the patient’s condition deteriorated to self-mutilation and muteness, leading to a diagnosis of schizophrenia. Following transfer to a new facility at which she received a complete neurological evaluation including CT scans, evoked potentials, lumbar punctures, brain biopsy of the right frontal lobe, EEGs, and an Amytal interview. The brain biopsy indicated some signs of encephalitis, but was considered inconclusive. The Amytal interview reflected decompensation and disorientation, a profile typical of encephalitis patients with neurological sequelae. It was only after these tests that the doctors re-considered the importance of the death of her horse from equine encephalitis shortly prior to the onset of her neuropsychological symptoms. The patient was eventually diagnosed with infectious encephalitis. With time, her speech returned, but her condition did not generally improve (Deaton et al., 1986). This unfortunate case exemplifies the need for early detection and treatment of encephalitis as well as rapid and proper diagnosis of sequelae. Today, we can only hope that improved detection standards for these viruses will aid in preventing such tragic outcomes.

### Eastern Equine Encephalitis

The first recorded epidemic of EEE was in horses of Massachusetts in 1831 (Hanson, 1957); however, the virus was not isolated and named until 1933 (Tenbroeck and Merrill, 1933). EEE is considered the most deadly of the three major encephalitic alphaviruses, with a case-fatality rate of 50–75% (CDC, 2010). It is therefore not surprising that the following case studies describe up to 90% of survivors as developing sequelae. In the United States alone, approximately 285 confirmed cases of EEE have been documented since 1964 (CDC, 2012, 2013). Of the 15 EEE cases reported in New Hampshire and Massachusetts children between 1970 and 2010, seven of these cases were associated with mental disability, with only four of those cases improving. Although these case reports do not describe the specific sequelae or categorize the mental disabilities encountered, it does provide us with incidence data and the potential frequency that neurological sequelae revert to normal (Silverman et al., 2013). Without specific treatment data, determination of the mechanisms that led to symptomatic improvement in these four cases is not possible.

Resulting from a fatal case of EEE, Reddy and colleagues in 2008 published autopsy findings that the patient had severe neuronal loss and gliosis of the dorsal motor nucleus that likely led to death from multiple organ failure (Reddy et al., 2008), as the dorsal motor nucleus is a key player in the parasympathetic system of the autonomic nervous system (Kumar et al., 2010). This study reinforces the necessity of autopsies performed on patients that succumb to viral encephalopathies to provide additional mechanistic information to be further studied in animal models.

A recent Panama outbreak of EEE/VEE in 2010 recorded 99 acute infection cases, where eight patients 1–13 years of age experienced neurological sequelae. The most common sequelae were seizures, which initially occurred in 63% of patients. Brain imaging was performed on these patients, and abnormalities were seen in the temporal lobe (Carrera et al., 2013). The temporal lobe is important for the establishment of and storage of new memories and their integration with emotion, language, and sensory processing (Kumar et al., 2010), thus providing a potential explanation for the types of neurological sequelae observed in this outbreak.

### Summary of Sequelae

As noted above, the severity of alphavirus-induced sequelae can vary from manageable learning disabilities to life altering and life threatening psychosis. Sequelae is most commonly described in children, but can affect individuals of any age group regardless of the severity of the febrile illness. The most detrimental cases of long term alphavirus sequelae seem to occur post encephalitis. WEEV has the most documented sequelae, with up the 90% of survivors developing long-term complication, while up to 75% of EEEV and 14% of VEEV survivors describe the same syndromes.

Interestingly, these sequelae are similar to what has been described for other arboviruses, such as the flaviviruses. Cognitive deficits and behavior changes are described for Japanese encephalitis virus (JEV) and West Nile Virus (WNV). Decreased memory and learning and behavior impairments have been reported post-JEV infection, with one study identifying that 2 years post infection 78% of survivors maintained memory and learning deficits and 47% still have behavioral impairments (Das and Basu, 2008; Hossain et al., 2010; Kakoti et al., 2013; Yin et al., 2015). In fact, a rat model of JEV replicated this memory and learning deficit post-infection (Chauhan et al., 2016). However, unlike in human cases, this deficit in the rat model was only transient.

West Nile Virus studies provide valuable information about neurological sequelae post-viral infections using more recent observational studies. Neurological sequelae of WNV are similar to what is described to JEV and the alphaviruses, with individuals experiencing cognitive and behavioral changes (Asnis et al., 2000; Madden, 2003; Burton et al., 2004; Klee et al., 2004; Gottfried et al., 2005; LaBeaud et al., 2006; Hart et al., 2014; Murray et al., 2014; Morjaria et al., 2015; Patel et al., 2015; Weatherhead et al., 2015; Anukumar et al., 2016; Winkelmann et al., 2016). Evaluations of the Houston West Nile Cohort described that even 8 years after infection, approximately 40% of survivors continued to experience some sort of sequelae (Murray et al., 2014; Weatherhead et al., 2015). Unlike the alphaviruses, WNV has also been described to lead to visual impairment and retinopathy, balance problems, hearing loss, and joint problems (Asnis et al., 2000; Burton et al., 2004; Gottfried et al., 2005; LaBeaud et al., 2006; McBride et al., 2006; Casetta et al., 2011; Hart et al., 2014; Murray et al., 2014; Weatherhead et al., 2015; Anukumar et al., 2016; Hasbun et al., 2016; Winkelmann et al., 2016).

### Economic Burden

Not only can these complications be detrimental to health, these conditions can lead to complex health economics. When Earnest et al. evaluated sequelae of the Texas WEE outbreak in 1971, they also determined the cost of infection per person at $320,000 (Earnest et al., 1971). More than twenty years later, Villari et al. (1995) determined the economic burden associated with patients suffering long-term sequelae due to EEE. Overall hospital costs for the first week of infection were determined by Villari and colleagues to be approximately $21,000. For someone suffering from long-term sequelae, medical costs would exceed $0.4 million per year per individual, with costs reaching up to $3 million in the life span of the affected individual. This estimate does not cover the cost of institutionalization for individuals that do not have dedicated caregivers. At the time this was calculated, the cost of insecticidal prevention of mosquito vectors ranged from $0.7 to $1.4 million dollars. Although large outbreaks of these diseases are uncommon today, the potential still exists and the economic consequences could prove disastrous.

## Applying Neuroscience to Viral Sequelae

As discussed above, long-term neurological sequelae are common among survivors of the three major encephalitic alphavirus infections. Neurological sequelae are those complications involving the brain that include cognitive, sensory, and motor deficits that may encompass emotional instability and seizure activity in the most severe cases. Unfortunately, little is known regarding susceptibility to, prevention of, or treatment for neurological sequelae resulting from alphavirus encephalitis. Studies using preclinical models are needed to establish mechanisms of infiltration or involvement of the CNS in neurological sequelae.

Well-established murine models are available to study each of these virus’ initial infection (Nalca et al., 2003; Paessler et al., 2004, 2006, 2007; Carrara et al., 2005; Steele and Twenhafel, 2010; Zacks and Paessler, 2010; Taylor et al., 2011, 2012) Behavioral platforms are also available to study neurological sequelae in mice, such as the SHIRPA battery for phenotypic assessment of neurological function, acoustic startle in the prepulse inhibition (PPI) paradigm, cued and contextual fear conditioning. Given the many years of clinical data in humans for each of these viruses and the murine models that closely mimic human diseases, we propose that neurological sequelae following acute viral infection can be detected in murine models for alphaviruses, providing a tool to study the mechanistic causes of these sequelae. We further propose that murine models can provide insights into the devastating neurological sequelae of life-threatening alphavirus encephalitis to identify therapeutic strategies to reverse or prevent post-acute-disease complications and improve the overall quality of life of survivors.

### SHIRPA

The SHIRPA battery for phenotypic assessment of neurological function is a general screen that quantitatively evaluates the animal’s behavior. The behavior is analyzed is three stages; the primary, secondary, and tertiary screens (Rogers et al., 1997). The primary screen is based on a screen developed by Irwin and colleagues in 1968 which evaluates defects in areas such as the animal’s gait, motor control, coordination, and muscle tone. These areas are scored with the SHIRPA protocol for quantitative analyses that can be compared over time and between groups. The secondary screen measures locomotor activity, food and water intake, balance and coordination, analgesia, histology, and biochemistry. Many of these tests are carried out with behavioral equipment, such as the rota-rod for balance and coordination, and do not require a scale rating for quantitative measures. These first two screens can be used to evaluate the phenotype for a wide range of applications, but the tertiary screen is designed to address neurological mutants. This final screen evaluates anxiety, learning and memory, PPI, electromyography, electroencephalography, nerve conduction, and magnetic resonance imaging (Rogers et al., 1997). The SHIRPA evaluation performed can be adapted to meet the needs of the study, and not all the steps described here are required. This is an excellent evaluation for use in preliminary evaluation of animal models for viral sequelae studies, as it can identify basic problems related to the brain and musculature without the need for expensive equipment. Additionally, many of the steps associated with SHIRPA are easily performed with little training and are easily adaptable to various biocontainment facilities with the use of proper animal handling techniques.

### Acoustic Startle Reflex (ASR)

**FIGURE 2:**
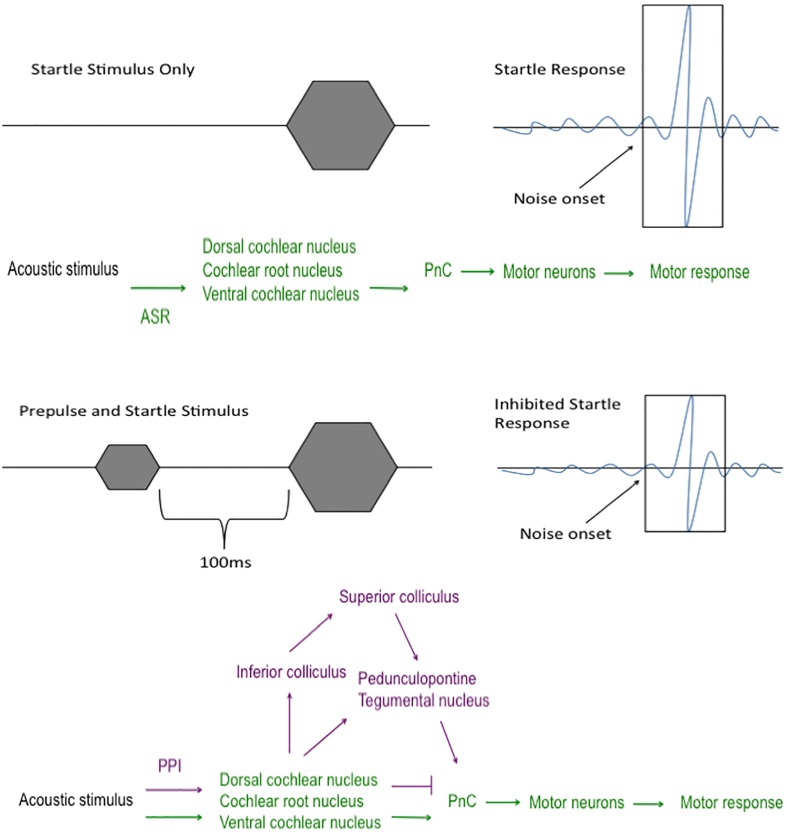
**Acoustic startle and prepulse inhibition pathways.** The pathway that the sound stimulus travels during acoustic startle reflex (ASR; green) and prepulse inhibition (PPI; purple). In the ASR circuit, the sound stimulus travels from the cochlear nuclei to the caudal pontine reticular nucleus (PnC) where it transmits the sound to the motor neurons and leads to a motor response, such as a jump or twitch. In the PPI circuit, a less intense acoustic stimulus, termed “prepulse”, is played within 100 ms prior to the startle stimulus. Since the portion of the ASR pathway (green) is preoccupied with the prepulse stimulus, the sound is forced to travel a different route (purple) to the PnC and motor neurons, which leads to a decreased motor response to the acoustic stimulus.

The startle response is an involuntary reaction elicited by an unexpected sound stimulus (Swerdlow et al., 1999). This reflex circuit begins in the cochlear nuclei of the brainstem where the acoustic startle stimulus is communicated to the caudal pontine reticular nucleus before impinging upon the motor neurons within the dorsal spinal cord to elicit a motor response (Davis et al., 1982b; Koch and Schnitzler, 1997; Koch, 1999; Fendt et al., 2001) (**Figure 2**). The acoustic startle threshold is the minimum decibel level that results in a flinch. Deaf animals do not flinch until the tone is 120 dB or above, likely responding to vibrations they sense through touching the equipment and not the sound itself (Crawley, 2007). Thus, intact hearing is crucial to successful measurements of both ASR and PPI; therefore, the SHIRPA evaluation is key to perform as an initial measure of neurological function. This is very important to keep in mind for viruses that may cause hearing loss or impairment on top of other neurological sequelae, such as West Nile virus (McBride et al., 2006; Casetta et al., 2011).

Several aspects of the startle response can be measured to determine changes in neurological processing and sensorimotor gating, such as latency of the response and the peak value of the motor response (Davis et al., 1982a,b). The latency is a measure of neural processing speed, or the time that it takes for the sound stimulus to elicit a motor response, while the peak value of the motor responses measures the intensity of the motor response. ASR has been used to study outcomes of infections with other disease. For example, infection with *Toxoplasma gondii* leads to a decreased-acoustic startle latency. *T. gondii* is an obligate intracellular protozoan that typically causes an asymptomatic infection in healthy individuals, but chronic infections leads to the development of cysts in the muscles and brain (Pearce et al., 2013). Evaluating these aspects of the startle response can provide a general overview of problems caused by encephalitic viruses post-infection.

### Prepulse Inhibition

Prepulse inhibition is an extension of the ASR that falls under the umbrella of sensorimotor gating paradigms. Basic PPI involves delivery of a low dB stimulus, the prepulse (between 3 and 12 dB above background), that is followed within 100 ms by a second higher db stimulus (**Figure 2**). Because of the temporal pairing of the prepulse with the main acoustic stimulus, the acoustic startle pathway is occupied leading to divergence of the sound stimulus through an alternate neural circuit that involves midbrain regions that results in feedback inhibition on motor neurons that decreases the motor response compared to the flinch response elicited in the absence of a prepulse (Kohl et al., 2013) (**Figure 2**). PPI deficits are well characterized in schizophrenias (Braff et al., 1978, 2001; Braff, 1992; Grillon et al., 1992; Kumari et al., 1999; Weike et al., 2000), obsessive compulsive disorder (Ahmari et al., 2012; Kohl et al., 2013) and Gilles de la Tourette’s syndrome (Castellanos et al., 1996; Swerdlow et al., 2001; Kohl et al., 2013). Patients with bipolar disorder also have detectable state-dependent PPI and sensorimotor gating deficits (Barrett et al., 2005; Rich et al., 2005; Carroll et al., 2007; Kohl et al., 2013). Several of these behavioral problems have been described in alphavirus survivors described above, as well as flavivirus infection, thus confirming the value of PPI as a tool to quantitatively measure the presence of neurological sequelae.

### Methods of Hearing Evaluation

In addition to the SHIRPA, a basic way to test hearing is with a clicker, a very small plastic box with a metal strip that makes a click sound when pressed. When testing hearing, a single click should result in an ear flick, jump, or brief head motionlessness (Crawley, 2007). Repeated clicks are considered ineffective. Should the ASR or PPI response change and the animal no longer responds normally to the clicker, additional tests should be done to evaluate the auditory threshold of the animal. These tests will be especially important to viral infections that are known to lead to hearing loss, such as WNV (McBride et al., 2006; Casetta et al., 2011; Anukumar et al., 2016). Two such tests are measurements of otoacoustic emissions (OAE) and auditory-evoked brainstem response (ABR). Conveniently, ASR, PPI, OAE, and ABR are tests available for both humans and rodent models, allowing for an easier transition into translational research.

## Conclusion

As we learn more about the basics of disease infection and proliferation, it is important to apply this knowledge and bring together techniques of other fields for a broader application of animal models to human disease. Currently, no one has evaluated the long-term sequelae of alphaviral encephalitis using the available animal models of acute infection. Exploration of cognitive changes in infected mice or another relevant rodent model has the potential to educate us about the sequelae in humans and allow to the delineation of mechanisms directly associated with sequelae. Utilizing preclinical models for alphavirus encephalitis and neurological sequelae requires BSL3-BSL4 facilities, which imposes logistic challenges for performing the types of exams and tests described above. These obstacles can be overcome with interdisciplinary training and assistance from environmental health and safety officers to develop a much needed small animal model. Such an animal model that allows for cognitive evaluation would also present an opportunity to study prevention of sequelae with vaccination, as the cost of a vaccine that prevents infection and long-term sequelae would outweigh the millions of dollars of medical resources required to treat one patient.

## Author Contributions

All authors listed, have made substantial, direct and intellectual contribution to the work, and approved it for publication.

## Conflict of Interest Statement

The authors declare that the research was conducted in the absence of any commercial or financial relationships that could be construed as a potential conflict of interest.
